# NtLTP4, a lipid transfer protein that enhances salt and drought stresses tolerance in *Nicotiana tabacum*

**DOI:** 10.1038/s41598-018-27274-8

**Published:** 2018-06-11

**Authors:** Yang Xu, Xinxin Zheng, Yunzhi Song, Lifei Zhu, Zipeng Yu, Liming Gan, Shumei Zhou, Hongmei Liu, Fujiang Wen, Changxiang Zhu

**Affiliations:** 1 State Key Laboratory of Crop Biology, College of Life Sciences, Shandong Agricultural University, Tai′an, Shandong P. R. China; 2 State Key Laboratory of Crop Biology, College of Agronomy, Shandong Agricultural University, Tai′an, Shandong P. R. China

## Abstract

Lipid transfer proteins (LTPs), a class of small, ubiquitous proteins, play critical roles in various environmental stresses. However, their precise biological functions remain unknown. Here we isolated an extracellular matrix-localised LTP, NtLTP4, from *Nicotiana tabacum*. The overexpression of *NtLTP4* in *N*. *tabacum* enhanced resistance to salt and drought stresses. Upon exposure to high salinity, *NtLTP4*-overexpressing lines (OE lines) accumulated low Na^+^ levels. Salt-responsive genes, including Na^+^/H^+^ exchangers (*NHX1*) and high-affinity K^+^ transporter1 (*HKT1*), were dramatically higher in OE lines than in wild-type lines. *NtLTP4* might regulate transcription levels of *NHX1* and *HKT1* to alleviate the toxicity of Na^+^. Interestingly, OE lines enhanced the tolerance of *N*. *tabacum* to drought stress by reducing the transpiration rate. Moreover, *NtLTP4* could increase reactive oxygen species (ROS)-scavenging enzyme activity and expression levels to scavenge excess ROS under drought and high salinity conditions. We used a two-hybrid yeast system and screened seven putative proteins that interact with NtLTP4 in tobacco. An MAPK member, wound-induced protein kinase, was confirmed to interact with NtLTP4 via co-immunoprecipitation and a firefly luciferase complementation imaging assay. Taken together, this is the first functional analysis of *NtLTP4*, and proves that *NtLTP4* positively regulates salt and drought stresses in *N*. *tabacum*.

## Introduction

Plants frequently encounter environmental stresses, such as salinity, drought, heavy metal, low temperature and microbial infections^1,2^. Among these stresses, salinity and drought stresses are major constraints on economic development and world food supplies^3^. Approximately 7% of the land area worldwide is affected by salinity and 40% is affected by drought^4,5^. The detrimental effects of salt and drought on plants are consequences of osmotic stress, ion imbalance and oxidative toxicity^6^. During the millions of years of evolution, plants have evolved to have high plasticity and elaborate mechanisms to protect themselves from salt and drought stresses. Plants prevent water loss while maximising water uptake to mitigate osmotic stress^7^. In addition, many protective proteins, including osmolytes, antioxidants and late embryogenesis abundant proteins, are produced and are likely to enhance stress resistance^8–10^. Another strategy for attaining improved salt tolerance is helping plants re-establish ionic homeostasis by using terminal determinants, such as salt overly sensitive (SOS) pathway components, Na^+^/H^+^ antiporter (NHX1) and high-affinity K^+^ transporter (HKT1)^11^. The stress-related molecular regulatory network is sophisticated and mostly unexplored. Thus, studying the key and novel genes that regulate salt or drought stress to cultivate the new varieties has become increasingly important for modern agriculture.

Lipid transfer proteins (LTPs) are extensively responsive to abiotic and biotic stresses^12^. Plant LTPs are a conserved class of small (7–10 kDa), abundant, ubiquitous and mostly basic proteins (isoelectric point usually 8.5–12). All examples of LTPs contain eight cysteine residues (C…C…CC…CXC…C…C) that form four conserved disulfide bridges that stabilise a bundle of four alpha-helices^13^. This bundle forms a hydrophobic pocket with a unique plasticity to accommodate a fatty acid, phospholipids or acyl-CoA^14,15^. Thus, they can exchange lipids between membranes *in vitro*^16^.

LTPs are divided into two families based on polypeptide length of mature protein. The first family of LTPs, named type I-LTP, possesses molecular masses of approximately 10 kDa and contains a signal peptide that is generally between 21–27 amino acids at the N-terminus^16^. The second family (type II-LTP) consists of peptides that possess molecular masses of approximately 7 kDa and contains a signal peptide between 27–35 amino acids^17^. The importance of these small proteins in plant growth and development has become increasingly clear, such as in cutin synthesis^16^, post-meiotic anther development^18^, and pathogen defence^19,20^. Moreover, LTPs are involved in abiotic stresses, the transcriptions of *CaLTP1* (*Capsicum annuum* L) and *TdLTP4* (*Triticum turgidum* L) are induced by ABA, ethylene, methyl jasmonic acid (MeJA) and salicylic acid (SA)^21,22^; Moreover, *ZmLTP3* and *AtLTP3* are positively regulated in salt and drought stresses, respectively^23,24^. Interestingly, LTP in tree tobacco increases cuticular wax accumulation to enhance drought resistance in a non-specific mechanism^25^. However, the molecular mechanism of LTPs involved in abiotic stress remains unclear.

Given their important roles in abiotic stress responses, LTPs have been studied in many plant species, including Arabidopsis (*Arabidopsis thaliana*), pepper (*Capsicum annuum* L), wheat (*Triticum turgidum* L) and maize (*Zea mays*)^21–24^. In the economic crop tobacco (*Nicotiana tabacum*), only NtLTP1 and NtLTP2 have been functionally characterised, and these are involved in plant defence responses and cell wall loosening mediation, respectively^19,26^. Therefore, illuminating the specific functions of other LTPs in tobacco is important.

In this study, *NtLTP4* was cloned from *N*. *tabacum*, which was dramatically induced by various stress treatments. Overexpression of *NtLTP4* in tobacco plants significantly enhanced salt and drought tolerance, which were caused by dramatically improved activities of reactive oxygen species (ROS)-scavenging enzymes. The analysis of the Na^+^ content further clarified the role of NtLTP4 by partially regulating the transcription levels of *NHX1* and *HKT1* to alleviate the toxicity of Na^+^. Moreover, NtLTP4 interacted with the tobacco MAPK wound-induced protein kinase (WIPK), which was associated with abiotic and biotic stress responses in tobaccoo^27,28^. Together, these data show the novel mechanism by which *NtLTP4* positively regulates salt and drought stresses.

## Results

### Characterisation of NtLTP4

**Figure 1:**
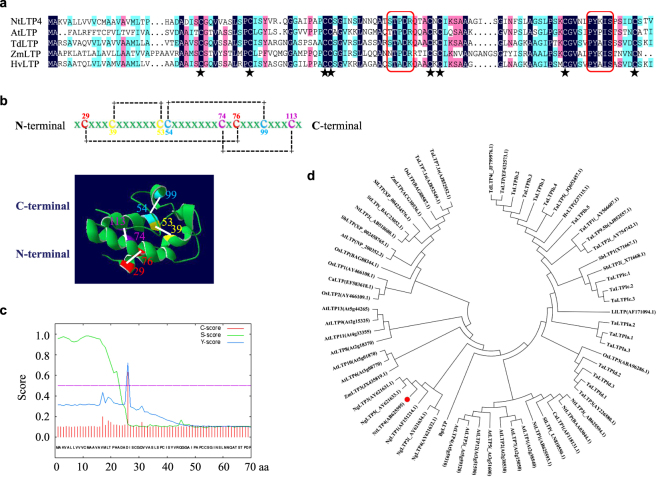
Characterisation of NtLTP4. **(a)** Amino acid sequence alignment of *N*. *tabacum* NtLTP4 with other LTPs. The sequence of NtLTP4 was 42% identity with the LTP of *Arabidopsis thaliana*, 55% identity with *T*. *durum*, 47% identity with *Zea mays* and 52% identity with *Hordeum vulgare*. At, *A*. *thaliana* (At5g59310); Td, *T*. *durum* (ABO28527.1); Zm, *Z*. *mays* (NP_001105392.1); Hv, *H*. *vulgare* (CAA85484.1). The conserved cysteine residues were highlighted with asterisks. Conserved and semi-conserved amino acid residues were represented by different colours. The consensus pentapeptides were highlighted in the red box. **(b)** Amino acid sequence and ribbon model of NtLTP4 structure showing the location of four pairs of disulfide bonds indicated by connecting lines. 3-D structural model of NtLTP4 predicted by SWISS-MODLE. **(c)** Predicted signal peptide of NtLTP4 (http://www.cbs.dtu.dk/services/SignalP/). **(d)** Phylogenetic tree of *N*. *tabacum* NtLTP4, highlighted by red circle. Nt, *N*. *tabacum* (AB625593.1, AB518680.1, AB625594.1); Td, *T*. *durum* (JF799976.1); Ta, *Triticum aestivum* (AJ852557.1, AJ852549.1, AJ852552.1); Hv, *H*. *vulgare* (CAA85484, CAA42832); Os, *Oryza sativa* (AY466108.1, AY466109.1); Zm, *Z*. *mays* (ACG30536.1); Si, *Setaria italica* (LN810550.1); At, *A*. *thaliana* (At2g38540, At5g59320, At5g59310, etc.); Ca, *C*. *annuum (*AF118131.1); Ng, *Nicotiana glauca* (AF151214.1); Sb, *Sorghum bicolor* (X71667.1, X71668.1, XP_002458765.1).

The full-length cDNA of *NtLTP4* was isolated from *N*. *tabacum* (AB625595.1). *NtLTP4* contained a 354 bp open-reading frame (ORF) and encoded a 117-amino acid polypeptide. Blast analyses showed that NtLTP4 shared approximately 50% identity with LTP proteins of other plants (Fig. 1a). Similar to other LTPs, NtLTP4 had a highly conserved motif that contained pentapeptides (T/S-X-X-D-R/K) or (P-Y-X-I-S). Furthermore, NtLTP4 possessed a unique and conserved eight-cysteine motif (29C-39C-53C-54C-74C-76C-99C-113C) that appeared to be a structural scaffold of conserved helical regions connected by variable loops (Fig. 1b). Moreover, NtLTP4 was predicted to possess a signal peptide at the N-terminus, which might be indispensable for localisation and exact function (Fig. 1c). A phylogenetic tree was constructed based on the amino acid sequence alignment of *N*. *tabacum* LTP4 with other LTPs (Fig. 1d). The proposed 3D structural model of NtLTP4 indicated a characteristic globular shape of orthogonal four a-helices and a hydrophobic core as typical LTPs in plants (Fig. 1b).

### Expression pattern analysis of NtLTP4

**Figure 2:**
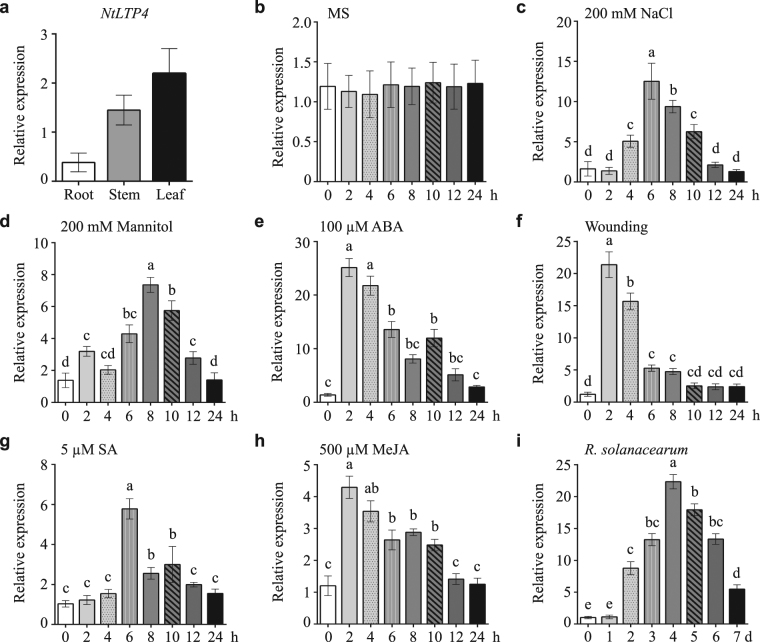
Expression pattern of *NtLTP4*. **(a)** Tissue-specific expressions of *NtLTP4* in roots, leaves and stems were quantified by qRT-PCR. **(b)** Total RNA extracted from the whole plants of 14-day-old *N*. *tabacum* without treatment and used for qRT-PCR. **(c–i)** Total RNA extracted from the whole plants of 14-day-old *N*. *tabacum* exposed to 200 mM NaCl, 200 mM mannitol, 100 μM ABA, wounding, 5 μM SA, 500 μM MeJA and *R*. *solanacearum* were used for qRT-PCR, respectively. Lanes represented the various durations of treatments, the internal control gene was *Actin*. Experiment was repeated three times. Error bars indicated SEM. One-way ANOVA (Duncan’s multiple range test) was performed, and statistical significant differences were indicated by different lowercase letters (*P* < 0.01).

*NtLTP4* was highly expressed in leaves and weakly expressed in roots, as observed by quantitative real-time polymerase chain reaction (qRT-PCR), which was similar to previous studies in 2012^19^ (Fig. 2a). The transcription levels of *NtLTP4* were measured after treatments representing drought (mannitol), salt (NaCl), wounding and *Rastonia*. *solanacearum*; and Murashige & Skoog (MS) treatment was used as control (Fig. 2b). A 12-fold induction of *NtLTP4* was observed at 6 h post-treatment with NaCl, and a 7.2-fold induction was observed with mannitol at 8 h (Fig. 2c,d). Furthermore, wounding and infection with *R*. *solanacearum* strongly induced the transcription of *NtLTP4* (Fig. 2f,i). In response to ABA, the *NtLTP4* transcription levels were strongly increased to 25-fold (Fig. 2e). Moreover, the *NtLTP4* transcription levels were enhanced by SA and JA by 5.7 and 4.3 times at 6 and 2 h, respectively (Fig. 2g,h). The rapid induction of *NtLTP4* under various stress treatments reveals that *NtLTP4* may be vital in responses to biotic or abiotic stresses in *N*. *tabacum*.

### NtLTP4 was localised to extracellular matrix

**Figure 3:**
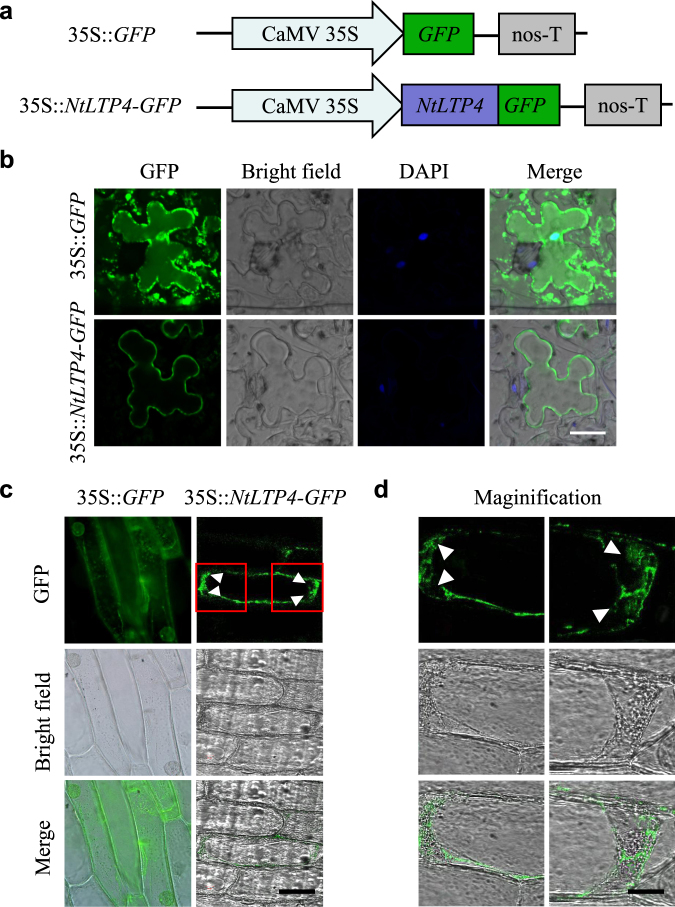
Subcellular localization analysis of the NtLTP4 in *N*. *benthamiana* leaves and onion epidermal cells. **(a)** Schematic illustration of the recombinant vector (35S::*NtLTP4-GFP*) and the control construct (35S::*GFP*). **(b)** Transient expression of NtLTP4-GFP and GFP in *N*. *benthamiana* leaves. Bars = 20 μm. **(c)** Transient expression of NtLTP4-GFP and GFP constructs in onion epidermal cells. GFP fluorescence visualised using fluorescence microscopy. Bar = 20 μm. **(d)** Images showing the magnified regions were indicated by red boxes in (c). Bar = 5 μm. Ten independent materials were analysed. The experiment was repeated with three biological replicates.

To investigate the subcellular localisation and potential biological functions of NtLTP4 in plants, the full-length ORF was cloned into the binary vector pROKII-GFP and resulted in translational fusion with green fluorescent protein (GFP) (Fig. 3a). The transient expressions of GFP-fused NtLTP4 proteins were analysed in *Agrobacterium*-infiltrated *Nicotiana benthamiana* leaves. Confocal fluorescence imaging revealed that NtLTP4-GFP fusion was primarily observed at cellular boundary (Fig. 3b). Subsequently, to examine whether the fluorescence derived from NtLTP4-GFP remained in the plasma membrane or was secreted to the cell wall, *Agrobacterium* that contained 35S::*NtLTP4-GFP* or 35S::*GFP* plasmids were introduced into onion epidermal cells. The onion epidermal cells that carried the 35S::*NtLTP4-GFP* plasmid emitted fluorescence only in the peripheral cell layers upon plasmolysis (Fig. 3c,d). The location can be explained by the presence of a potential signal peptide in NtLTP4, as predicted in Fig. 1c, that drives LTPs into the extracellular matrix. This result may be linked to the cell-to-cell signal transduction function.

### Overexpression of NtLTP4 enhanced salt tolerance in transgenic tobacco and yeast

*NtLTP4* was highly induced by various abiotic stresses, thereby suggesting its role in multiple stress responses, especially to salt stress. To understand further the molecular mechanisms underlying the stress responses of *NtLTP4*, we produced *NtLTP4*-overexpressing transgenic tobacco plants. qRT-PCR analysis was conducted to detect the expression levels of *NtLTP4* in different overexpressing lines (OE) (Supplementary Fig. S1). The transgenic lines with different expression levels were shown in Supplementary Fig. S1, and OE1 (high), OE2 (medium) and OE3 (low) were chosen for in-depth physiological analysis.

**Figure 4:**
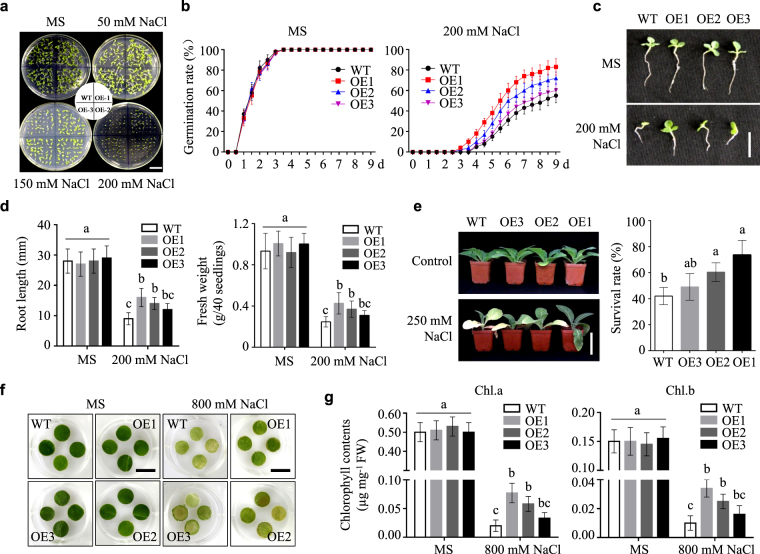
Overexpression of *NtLTP4* in transgenic plants enhanced salt tolerance. **(a)** Seeds sown on MS medium containing various concentrations of NaCl. Bar = 1.5 cm. **(b)** Germination rates of WT and OE lines with or without 200 mM NaCl treatment. **(c)** Seedling phenotype of 14-day-old WT and OE lines with or without salt stress treatment. Bar = 1 cm. **(d)** Root length and fresh weight of seedlings shown in (**c**). **(e)** Phenotype and survival rates of 8-week-old WT and OE plants grew in soil irrigated with water and 250 mM NaCl solution for 1 month. Bar = 2 cm. **(f)** Representative phenotypes of leaf disks from detached leaves of WT and OE plants with MS supplied with or without 800 mM NaCl. Bar = 1 cm. **(g)** Quantification of chlorophyll contents in different plants. Chl.a = chlorophyll a. Chl.b = chlorophyll b. Data were mean values of three biological repeats. Error bars indicated SEM. One-way ANOVA (Duncan’s multiple range test) was performed, and statistical significant differences were indicated by different lowercase letters (*P* < 0.01).

To investigate possible effects on salt stress, wild-type (WT) and OE lines on MS agar medium were germinated with or without NaCl. No obvious differences were observed between WT and OE lines under standard growth conditions, as illustrated in Fig. 4a,b. However, the germination of OE lines was more insensitive than that of WT upon exposure to high salinity. Only 50% of WT seeds were germinated; however, 55% of OE3, 65% of OE2 and 80% of OE1 seeds were germinated in 200 mM NaCl (Fig. 4b). Moreover, we tested whether *NtLTP4* played a role in seedling establishment. *N*. *tabacum* seedlings were transferred to MS medium with or without NaCl. The WT showed significantly enhanced growth inhibition compared with those of OE lines (Fig. 4c). The primary root lengths of OE1, OE2 and OE3 lines were increased by 8, 7 and 4 mm, respectively, compared with those of WT in the presence of 200 mM NaCl (Fig. 4c,d). In addition, transgenic seedlings with NtLTP4 indicated up to 18–50% significantly higher fresh weight than the wild-type at 200 mM NaCl (Fig. 4d). To study further the function of *NtLTP4* in the vegetative stage, 8-week-old WT and OE plants were irrigated with salt solution for 1 month. The WT plants indicated severe growth retardation, leaf curling and chlorosis compared with the OE lines (Fig. 4e). Furthermore, approximately 48–75% of OE plants survived under high salinity conditions, whereas only 40% of WT plants survived (Fig. 4e).

As shown in Fig. 4f, no deformities were observed in all MS-treated plants for 3 d. The leaf disks of WT plants indicted severe signs of bleaching or chlorosis compared with those of OE lines after the leaf disks were floated in salt solution. This result was further confirmed by measuring the leaf disk chlorophyll contents with or without NaCl treatment (Fig. 4g).

A yeast system was used to further verify the function of *NtLTP4* in response to salt stress, and pYES2-*NtLTP4*, pYES2-*NtLTP4*^27–118,*aa*^ (truncated without signal peptide) and pYES2 empty vectors were transformed into yeast (Gold Yeast, Clontech). The corresponding transformants were spotted on YPDA solid medium with or without NaCl. All the transformants indicated severe growth inhibition on the YPDA medium with NaCl (Supplementary Fig. 2a). The transgenic yeast that overexpressed *NtLTP4* exhibited enhanced salt tolerance resistance; however, the pYES2-*NtLTP4*^27–118,*aa*^ transformants indicated same growth as the empty vector control (Supplementary Fig. 2a). Then, the transgenic strains were inoculated into the YPDA liquid medium that contained different NaCl concentrations, and absorbance of the stock cultures were determined 48 h later. The absorbance of the yeast strain that contained *NtLTP4* was higher than those of the *NtLTP4*^27–118,*aa*^ and empty vector control (Supplementary Fig. 2b). In summary, *NtLTP4* enhanced the salt stress tolerance in yeast, and the potential signal peptide might be indispensable to the involvement of *NtLTP4* genes in abiotic stress responses.

These results indicate that *NtLTP4* overexpression in tobacco and yeast increase salt stress tolerance.

### *NtLTP4* regulated Na^+^ homeostasis by *HKT1* and *NHX1*

**Figure 5:**
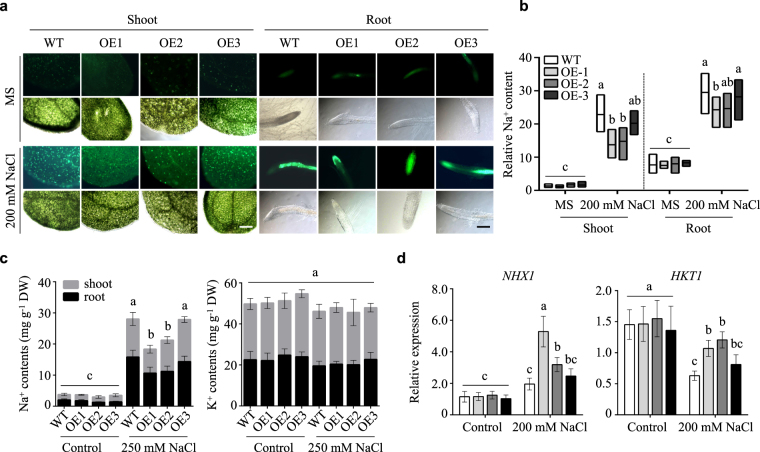
*NtLTP4* regulated Na^+^ homeostasis by controlling the expression levels of *HKT1* and *NHX1*. **(a)** Na^+^ contents in 14-day-old seedling shoots and roots of WT and OE lines treated with MS or 200 mM NaCl for 48 h exhibited by CoroNa™ Green dye. Green-fluorescence detected by confocal microscopy. Ten independent plants used in detection. Bar = 40 μm. **(b)** Na^+^ content quantitatively analysed by ImageJ. **(c)** Contents of Na^+^ and K^+^ in shoots and roots of 8-week-old plants irrigated with water or 250 mM NaCl for one month and measured by atomic absorption spectrometry. **(d)** Relative expression analysis of *NHX1 and HKT1* in 14-day-old WT and OE lines treated with MS or 200 mM NaCl for 48 h. Data as mean values of three biological repeats. Error bars indicated SEM. One-way ANOVA (Duncan’s multiple range test) was performed, and statistical significant differences were indicated by different lowercase letters (*P* < 0.01).

To understand the physiological role of *NtLTP4* in salt stress response, CoroNa™ Green dye, a green fluorescent indicator of Na^+^, was used to specifically measure the Na^+^ contents in 14-day-old seedlings. Confocal microscopy revealed intense fluorescence signals in leaves and root tips of WT lines compared with those in transgenic plants after salt treatment, and the fluorescence in leaves of OE lines was barely detectable (Fig. 5a,b). To confirm the above results, Na^+^ levels in the shoots and roots were measured separately using atomic absorption spectrometry. OE lines contained lower concentrations of Na^+^ than WT plants treated with 250 mM NaCl, and statistically significant differences were observed mainly in the shoots (Fig. 5c). However, the K^+^ contents remained fairly similar among the Arabidopsis samples with or without salt treatment (Fig. 5c). Therefore, the salt-tolerant phenotype of OE lines may be caused by the reduced levels of Na^+^ contents, thereby suggesting that *NtLTP4* may regulate Na^+^ homeostasis in plants.

Na^+^ homeostasis is mainly mediated by Na^+^ transporters, including salt overly sensitive 1 (SOS1), SOS2, SOS3, Na^+^ transporter HKT1 and vacuolar membrane Na^+^/H^+^ exchangers NHX1. In addition, vacuolar membrane H^+^-pyrophosphatase (VP), vacuolar-type pyrophosphate-energised membrane proton pump (MVP) and high-affinity K^+^ transporters (HAK) are implicated in salt stress^1^. The sequences of salt stress-related Na^+^ transporters from *N*. *tabacum* genome website (https://solgenomics.net/organism/Nicotiana_tabacum/genome) were used to design primers for qRT-PCR assay. No obvious differences were observed in the expression levels of *NtSOS1*, *NtSOS2*, *NtSOS3*, *NtVP1*, *NtHAK1* and *NtHAK8* between OE and WT lines (Fig. 5d; Supplementary Fig. S3). Under saline conditions, the expression levels of *NtHKT1* and *NtNHX1* in OE lines were relatively higher compared with those in WT plants, and these transporters have been reported to be vital salt-tolerance determinants (Fig. 5d). Arabidopsis plants that overexpress *HKT1* and *NHX1* have been reported to enhance salt tolerance^29,30^. Therefore, *NtLTP4* plays a role in tobacco at least partially by indirectly regulating the transcription of transporters, such as *NtHKT1* and *NtNHX1*, to maintain Na^+^ homeostasis.

### Overexpression of *NtLTP4* enhanced drought tolerance in transgenic tobacco and yeast

**Figure 6:**
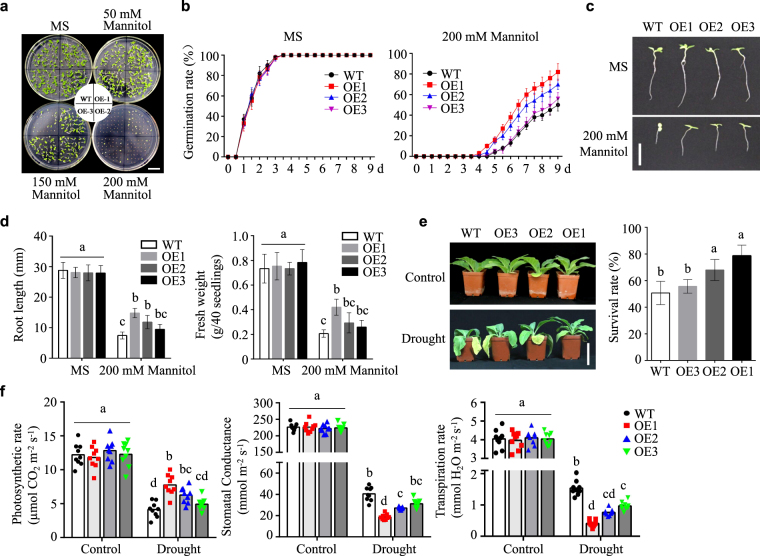
Overexpression of *NtLTP4* in transgenic plants enhanced drought tolerance. **(a)** Seed germination on MS medium containing various concentrations of mannitol. Bar = 1 cm. **(b)** Germination rates of WT and OE lines in MS medium with or without 200 mM mannitol. **(c)** Seedling phenotype of 14-day-old WT and OE lines with or without mannitol treatment. Bar = 1 cm. **(d)** Root length and fresh weight of seedlings shown in (**c**). **(e)** Phenotype and survival rate of 8-week-old WT and OE plants grew in soil with or without dehydration treatment for 15 days. Bar = 2 cm. **(f)** Photosynthetic performances of WT and OE plants with or without drought treatment. Pn: net photosynthetic rate (Pn, μmol CO_2_ m^−2^s^−1^) Gs: stomatal conductance (gs, mmol H_2_O m^−2^s^−1^) E: transpiration rate (E, mmol H_2_O m^−2^s^−1^). Data as mean values of three biological repeats. Error bars indicated SEM. One-way ANOVA (Duncan′s multiple range test) was performed, and statistical significant differences were indicated by different lowercase letters (*P* < 0.01).

To further investigate whether *NtLTP4* functions in drought stress, seeds of WT and OE lines were evaluated by using MS medium supplemented with exogenous mannitol and PEG (Fig. 6a; Supplementary Fig. S4). The germination rate of WT plants was approximately 45% and those of OE lines were approximately 52–78% (OE1 78%, OE2 65%, OE3 52%) in the presence of 200 mM mannitol (Fig. 6b). In order to explore whether *NtLTP4* functions at the seedling stage, root growth inhibition and fresh weight were measured on vertical growth plates with different concentrations of mannitol. Three OE lines indicated enhanced drought stress resistance compared with WT plants, regardless of root elongation or fresh weight (Fig. 6c,d). Moreover, similar phenotypes were observed on PEG treatment at germination and seedling stage (Supplementary Fig. S4).

To further assess the effect of drought stress on the growth of *NtLTP4* transgenic lines during vegetative growth stage, 8-week-old WT and OE plants were grown with or without water for another 15 d. The WT plants showed worse leaf wilting and chlorosis than OE plants under drought treatment (Fig. 6e). Upon re-watering, the survival rate of transgenic plants reached approximately 55–80%, whereas WT plants showed a survival rate of 50% (Fig. 6e). Additionally, we evaluated the effects of drought stress on photosynthetic gas exchange parameters. As shown in Fig. 6f, no significant differences were observed in net photosynthesis rates (Pn) between transgenic and WT plants under standard conditions. Drought stress resulted in marked decrease in Pn in transgenic and WT plants, whereas this value was maintained at a higher level in OE lines than in WT under drought stress (Fig. 6f). The transpiration rates (Tr) of the transgenic and WT plants decreased after drought treatment, whereas the OE lines indicated lower transpiration rates than in WT plants (Fig. 6f). Similar results were observed for stomatal conductance (Fig. 6f). These results indicated that the *NtLTP4*-overexpressing lines were more tolerant to drought stress than WT plants secondary to regulation of transpiration rates and stomatal conductance.

The above results, in which overexpression of *NtLTP4* enhanced drought tolerance, were confirmed in the yeast system treated with mannitol instead of NaCl, as described in result 4 (Supplementary Fig. S2). These data indicate that transgenic tobacco plants and yeast strains that overexpressed *NtLTP4* demonstrate enhanced drought tolerance when subject to deficient water supply.

### Overexpression of *NtLTP4* enhanced tolerance to oxidative stress in transgenic plants

**Figure 7:**
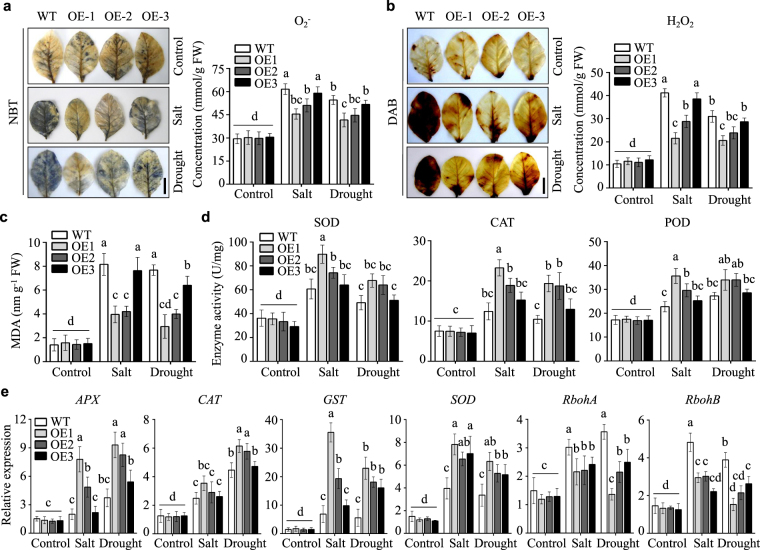
Overexpression of *NtLTP4* in transgenic plants decreased the ROS production and oxidative damage under salt and drought treatments. **(a)** Photographs of NBT staining and O_2_^−^ concentrations measured by kit in WT and OE plants after drought and salt treatments. Bar = 2 cm. **(b)** Photographs of DAB staining and H_2_O_2_ concentrations measured by kit in WT and OE plants after drought and salt treatments. Bar = 2 cm. **(c)** MDA contents in WT and OE plants after salt and drought treatments. **(d)** SOD, POD and CAT activities measured by corresponding kit, respectively. **(e)** The expression levels of ROS-scavenging or ROS-producing genes *APX*, *CAT*, *GST*, *SOD*, *RbohA* and *RbohB* in WT and OE lines. Data as mean values of three biological repeats. Error bars indicated SEM. One-way ANOVA (Duncan′s multiple range test) was performed, and statistical significant differences were indicated by different lowercase letters (*P* < 0.01).

Abiotic stresses generally enhance the production of cellular ROS and cause oxidative damage^31^. To investigate whether *NtLTP4* regulates ROS accumulation, 3,3′- diaminobenzidine (DAB) and nitro blue tetrazolium (NBT) staining were applied to evaluate the levels of two prominent ROS species, namely, H_2_O_2_ and O_2_^−^, in leaves that were detached from WT and transgenic plants after being subjected to salt and drought stresses. As shown in Fig. 7a,b, no difference was observed between OE and WT lines under normal conditions. However, DAB and NBT staining were stronger in WT than in OE lines after salt and drought treatments, and accumulation of H_2_O_2_ and O_2_^−^ in WT plants were significantly elevated (Fig. 7a,b). Malondialdehyde (MDA) is the final product of lipid peroxidation, and the MDA contents in OE lines were lower than in WT plants, thereby indicating that the transgenic plants suffered slighter membrane damage (Fig. 7c).

Therefore, plants evolve antioxidant systems for regulating intracellular ROS homeostasis^31,32^. The activities of three prominent antioxidant enzymes, namely, superoxide dismutase (SOD), peroxidase (POD) and catalase (CAT), were monitored before and after treatment, and no differences were found under normal growth conditions. After salt and drought treatments, SOD, CAT and POD activities were significantly higher in OE than in WT plants (Fig. 7d). Furthermore, the mRNA levels of several important ROS-scavenging enzymes encoding genes, such as *SOD*, *APX*, *CAT* and glutathione S-transferase (*GST*) and ROS-generating related gene, including respiratory burst oxidase homolog genes (*RbohA* and *RbohB*) were determined by qRT-PCR analysis. After salt and drought treatments, the expression levels of *SOD*, *APX*, *CAT* and *GST* dramatically increased in OE lines to levels higher than those in WT plants (Fig. 7e). By contrast, the expression levels of *RbohA* and *RbohB* were markedly decreased (Fig. 7e). These results suggest that *NtLTP4* may improve ROS scavenging and suppress ROS production to relieve ROS toxicity caused by salt and drought stresses.

### NtLTP4 interacted with WIPK

**Figure 8:**
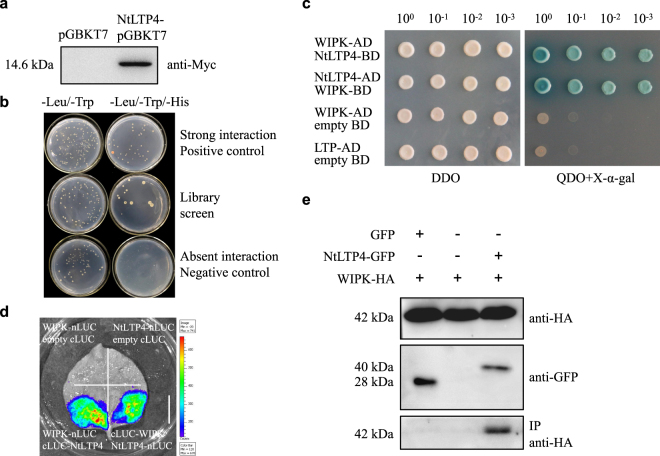
Interaction of NtLTP4 with WIPK. **(a)** Detection of pGBKT7-NtLTP4-Myc bait fusion protein via Western Blot. Lane 1: pGBKT7 empty insert (negative control), lane 2: NtLTP4-pGBKT7-Myc. **(b)** Yeast two-hybrid screening assay was conducted in yeast. pGBKT7-p53 and pGADT7-T were used as positive control, and pGBKT7-pLam and pGADT7-T were used as negative control. **(c)** NtLTP4 interacted with WIPK by yeast-two-hybrid. Bait fusion vector and prey fusion vector pGBKT7-NtLTP4/pGADT7-WIPK and pGBKT7-WIPK/pGADT7-NtLTP4 were transformed into the yeast strain Gold Yeast. **(d)** NtLTP4 interacted with WIPK by LCI assay. Bar = 2 cm, **(e)** The interaction between NtLTP4 and WIPK was tested via co-immunoprecipitation (Co-IP) assay of transiently overexpressed WIPK-HA and NtLTP4-GFP in *N*. *benthamiana* leaves. *N*. *benthamiana* leaves that co-expressed GFP and WIPK-HA were used as negative controls.

To further determine the mechanism by which *NtLTP4* positively regulated salt and drought stresses in tobacco, we initially used yeast two-hybrid screening assay to investigate the candidate proteins that physically interact with NtLTP4 (Fig. 8a,b). Bait fusion vector (pGBKT7-NtLTP4) was constructed and transformed into the yeast strain Yeast Gold. Western Blot analysis with Myc monoclonal antibody was conducted to confirm the fusion protein of 14.6 kDa (11 kDa NtLTP4 + 3.6 kDa Myc fusion = 14.6 kDa) in yeast (Fig. 8a). We identified seven putative interactors, and stress-related WIPK was selected for further study (Supplementary Table S1).

The interaction between NtLTP4 and WIPK in yeast was confirmed by yeast two-hybrid assay (Fig. 8c). Then, the possible interaction between NtLTP4 and WIPK was further tested via a firefly luciferase complementation imaging (LCI) assay. As shown in Fig. 8d, coexpression of cLUC-WIPK and NtLTP4-nLUC or cLUC-NtLTP4 and WIPK-nLUC in *N*. *benthamiana* leaves strongly complemented the LUC activity compared to the negative control. Moreover, co-immunoprecipitation (Co-IP) assay of transiently overexpressed WIPK-HA and NtLTP4-GFP in *N*. *benthamiana* leaves was also performed. Total proteins were extracted and immunoprecipitated by using specific GFP-bead mixture, and then the proteins were analysed by Western Blot assay using an HA antibody. The results showed that WIPK was immunoprecipitated with NtLTP4 but not GFP (Fig. 8e). WIPK has been reported to be related to abiotic and biotic stress responses in previous studies^27,33^. Given that NtLTP4 is capable of interacting with WIPK, we conclude that *NtLTP4* functions in salt and drought stress is associated with WIPK.

## Discussion

LTPs are abundant, small and lipid-binding proteins that are involved in various physiological processes in plants, including cutin synthesis, somatic embryogenesis, post-meiotic anther development, pathogen defence and long-distance signalling of systemic acquired resistance (SAR)^16,18–20,34,35^. Moreover, LTPs can be implicated in abiotic stresses in various species, such as drought, cold and salt stresses^25,36,37^. In the present study, we identified a novel LTP protein from tobacco, *NtLTP4*, which was dramatically induced and upregulated rapidly by various abiotic stresses (Fig. 2). Furthermore, the *NtLTP4*-overexpressing tobacco plants enhanced tolerance to high salinity and drought stresses (Figs 4 and 6). The Na^+^ content analysis assay further clarified the role of *NtLTP4* by partially regulating the transcription levels of *NHX1* and *HKT1* to alleviate the toxicity of Na^+^ (Fig. 5). In addition, *NtLTP4* could reduce transpiration rate to reduce water loss rate (Fig. 6f). Interestingly, NtLTP4 was found to interact with stress-related MAPK, WIPK in plants (Fig. 8). Thus, the hyposensitivity phenotypes of *NtLTP4*-overexpressing tobacco plants might be associated with WIPK-mediated stress response.

Plants that expressed a mutant form of *Defective in induced resistance 1* (*DIR1*), an apoplastic *LTP*, demonstrate normal local resistance to pathogens but fail to develop SAR or express pathogenesis-related proteins in their systemic leaves^35^. This observation suggests that DIR1 may function as a mobile SAR signal that transmits the information from inoculated leaves to distant leaves^35^. Moreover, induced SAR in grafted scion leaves reveals that CALTP also plays a role in long-distance systemic signalling in plants^38^. In our study, NtLTP4 was localised to the extracellular matrix (Fig. 3c), thereby suggesting that the stress-response LTP might also act as signal molecular involved in long-distance signalling transduction in tobacco. NtLTP4 was highly expressed in leaves and stems but weakly expressed in roots (Fig. 2a). However, Na^+^ was absorbed in large amounts by roots, and NtLTP4 might be transported from shoot to root via the phloem for roles in the latter. Future studies will test this hypothesis via grafted scion assay.

High salinity is a serious factor that seriously reduces crop yield, thereby inducing Na^+^/K^+^ imbalance and ion toxic effects. Plants have evolved high plasticity and elaborate mechanisms to adapt to ionic stress typically involve the removal of Na^+^ from root via the SOS1 Na^+^/H^+^ antiporter, the removal of Na^+^ from transpiration stream by HKT transporters and the vacuolar sequestration of Na^+^ via NHX1 tonoplast antiporter^39^. Na^+^ accumulation was lower in OE lines compared with those of WT lines after salt treatment (Fig. 5a–c), thereby suggesting that NtLTP4 could maintain Na^+^ homeostasis in plants. The expression levels of *SOS1*, *SOS2*, *SOS3*, *HAK1*, *HAK8*, *MVP3* and *VP1* were almost the same in OE lines and WT with or without salt treatment, whereas the OE lines obviously accumulated more transcripts of *NtNHX1* and *NtHKT1* (Fig. 5d; Supplementary Fig. S3). These results suggested that the salt tolerance phenotype of OE lines was not caused by the induction of Na^+^ efflux from root epidermis or regulation of K^+^ and H^+^ equilibrium but probably by compartmentalization of Na^+^ into vacuoles via NHX1 and removal of Na^+^ from transpiration stream by HKT1 in tobacco.

Salt and drought-induced secondary messengers activate diverse signalling proteins, including various members of MAPK family. MAPK3 is strongly associated with stress signalling, including salt and drought in Arabidopsis^40^. In Arabidopsis, MAPK3 seems to regulate the activity of heat shock factor HSFA4A or form complex with ZAT10, thereby conferring tolerance to salt stress^41,42^. Moreover, MAPK3 enhances tolerance to drought stress via drought-induced stomatal closure^43^. To date, LTPs have not been directly connected with MAPKs in tobacco. Previous study has implied that a LTP-related Hybrid proline-rich protein (HyPRP) acts as a direct component in the MAPK-mediated salt stress response in Arabidopsis^44^. In this study, NtLTP4 was detected as a direct interactor of WIPK for the first time (Fig. 8). WIPK, a homolog of MAPK3, was also reported to regulate the abiotic stresses in tobacco^33^. Thus, NtLTP4 that mediates salt and drought stress signalling in tobacco is at least linked with WIPK-related stress response. However, how NtLTP4-WIPK association is involved in abiotic stress remains obscure. Whether NtLTP4 can be phosphorylated by WIPK must also be tested via *in vitro* and *in vivo* kinase assays.

Tobacco LTP1-JA can bind to specific plasma membrane sites to activate plant defence, characterized as elicitin receptors^45^. Elicitins are small, cysteine-rich and lipid-binding proteins secreted by *Phytophthora*^46^, and they share some structural and functional properties with LTPs. The elicitin receptor has been assumed to be the allosteric calcium channel by isotopic tracing technique^46,47^. In addition, Ca^2+^ receptor, calmodulin (CaM)-binding site is found within the 69–80 amino acids of the C-terminal region of LTP1, thereby indicating that LTPs may be a new family of CaM-binding proteins that are involved in the CaM-mediated signalling pathway^48,49^. The Ca^2+^/CaM complex are evoked in response to different stimuli such as drought, salt stress, light and plant hormones, which represent intricate signalling networks involved in various abiotic stresses^50^. Ca^2+^/CaM complex have been reported to regulate the Na^+^ determiner NHX1, activate MAPK cascade pathway and induce stomatal closure in response to abiotic and biotic stresses^51–53^. All of the studies at least indirectly link NtLTP4 with Ca^2+^ signalling pathway and support the assumption that NtLTP4 functions in salt and drought stress in a Ca^2+^-dependent manner. Thus, we can hypothesise that salt and drought stresses elicit an increase in cytosolic Ca^2+^ and that CaM may detect the stress signal and then bind to NtLTP4 and evoke downstream responses in plants.

In summary, our findings provided the first evidence that *NtLTP4* enhanced salt and drought tolerance in transgenic tobacco by alleviating Na^+^ and ROS toxic effects and regulating transpiration rate and stomatal conduction. Moreover, our study implied LTP as a direct interactor in the MAPK-mediated stress response in tobacco. Our results provided insight into the association of plant abiotic stress response and LTPs. To further understand the function of NtLTP4 in MAPK cascade pathway, we must clarify the physiological function of the WIPK-NtLTP4 complexes in tobacco.

## Materials and Methods

### Plant materials and growth conditions

*N*. *tabacum* seeds were germinated on MS medium and transferred to a growth chamber with a 16 h/8 h light/dark cycle at 28 °C. Plants were sprayed with 100 μM ABA, 500 μM MeJA or 5 μM SA after 14 days of growth. For the salt and drought treatments, the seedlings were transferred to MS medium with 200 mM NaCl or 200 mM mannitol. The seedlings were cut using scissors to mimic wounding and immersed into *R*. *solanacearum* suspension for biotic stress treatment. Plants grown under the normal condition were used as control.

### RNA extraction and qRT-PCR assay

RNA was extracted from various issues or 14-day-old seedlings after different treatments using TRIzol reagent (Invitrogen, Carlsbad, CA, USA) according to manufacturer’s instructions. Total RNA was digested to remove the genomic DNA and used for first-strand cDNA synthesis using reverse transcriptase (TIANGEN). qRT-PCR was performed using SYBR Premix Ex Taq (TaKaRa, Dalian, China) and CFX96TM Real-Time PCR Detection System (Bio-Rad, Hercules, CA, USA) to examine gene expression levels. The housekeeping gene *Actin* in *N*. *tabacum* was used as control. The expression levels were calculated using the 2^−ΔΔCt^ comparative CT method. Three biological replicates were performed.

### Sequence analysis

The sequence of *NtLTP4* (AB625595.1) was obtained from National Center of Biotechnology Information (NCBI). The software ClustalW (1.82) from Europe Biotechnology Information was employed for the multiple sequence alignment of amino acid sequences of NtLTP4 with LTPs from various plants retrieved from NCBI. Subsequently, a phylogenetic tree was constructed by neighbour-joining (NJ) method using molecular evolutionary genetics analysis and sequence alignment tool MEGA 3.1. The reliability of the tree was measured using bootstrap analysis with 1500 trials. The theoretical calculation of molecular weight was analysed with ExPASy using Compute pI/Mw tool (http://www.expasy.ch/tools/pi_tool.html). For molecular modelling, SWISS-MODEL was used to predict the 3-D structure of NtLTP4.

### Subcellular localisation of NtLTP4

To construct the 35S::*NtLTP4-GFP* expression plasmid, the CDS of *NtLTP4* was inserted into the binary vector pROKII-GFP. Both 35S::*GFP* and the recombinant vectors were transferred into *N*. *benthamiana* and onion epidermal cells. The onion tissue was soaked in a 0.3% sucrose solution for 5 minutes. Fluorescence was observed using a fluorescence microscope (LSM 510 META, ZEISS, Germany). Ten independent plants were analysed. The experiment was repeated with three biological replicates.

### Construction and genetic transformation

The full-length coding region of *NtLTP4* was inserted into the binary vector pROKII. The vector was then electroporated into *Agrobacterium tumefaciens* (strain LBA4404) using a micropulser (BioRad, Hercules, CA, USA).

The *A*. *tumefaciens*-mediated leaf disc method was used to transform *N*. *tabacum* to achieve transgenic tobacco plants^54^. The infected leaf discs were placed in a selection medium composed of MS medium supplemented with 100 mg/L kanamycin, and transformations were confirmed using PCR. The seeds harvested from regenerated *T*_0_ plants were reproduced to produce the *T*_1_ and *T*_2_ generation plants for kanamycin resistance and confirmed as homozygotes using PCR.

### Quantification of seed germination and root length

The *N*. *tabacum* seeds of each genotype were plated on MS medium containing 1% (w/v) sucrose with various concentrations of NaCl, mannitol or PEG. Seed-dotted plates were maintained in the dark at 4 °C for 3 days and transferred to a growth chamber with their germination percentages measured daily. Germination assays were done with three replicates of 120 seeds.

The 3-day-old seedlings that were sown on MS medium were transferred to MS medium with various concentrations of NaCl, mannitol or PEG. All seedlings were placed vertically in growth chamber. Root elongation and fresh weight were evaluated in the 14-day-old seedlings.

### Salt stress treatment

The 8-week-old plants of WT and OE lines were irrigated with or without 250 mM NaCl solution for one month and survival rate was recorded. Roots and shoots were collected separately and enough materials were dried in an oven at 65 °C for 4 days, then were extracted with 16 M H_2_SO_4_ and heated to 300 °C for 8 hours. The Na^+^ and K^+^ contents of the supernatants were analysed by Atomic Abserption Spectrometer (Analytik Jena nevAA330) using a flame emission method.

For Na^+^ dying assay, the 14-day-old plants of WT and OE lines sown on MS medium were transferred to MS medium with or without 200 mM NaCl for 48 h. The CoroNa™ Green dye (Life, USA), which exhibited a fluorescence emission upon binding Na^+^, was used to specifically determine the contents of Na^+^. Seedlings were incubated in Na^+^ dye for 2 hours at 37 °C. Confocal laser scanning fluorescence microscopy (LSFM, Zeiss) was used to observe the fluorescence of root tips and leaves of seedlings. Ten independent plants were analysed. The experiment was repeated with three biological replicates.

### Drought stress treatment

The 8-week-old seedlings of WT and OE lines were held without water for 15 days. The plants were then re-watered for 3 days and survival rate was recorded. In another experiment, 8-week-old WT and OE plants were held without water for 15 days, then the net photosynthetic rate (Pn), transpiration rate (E) and stomatal conductance (Gs) were measured by a portable photosynthetic system (CIRAS-3, PP Systems, Hitchin, UK) in the morning between 9:00–11:00. Photosynthesis was measured indoors. CO_2_ concentration was controlled at 380 ppm. Light intensity was controlled at 1000 μmolm^−2^·s^−1^ PPFD and relative humidity ranged from 60–70%. The temperature inside the leaf chamber was 25 °C.

### Estimation of Chlorophyll Content

Leaf disc assay was performed. A hole-punch was used to punch similar-sized disks from 8-week-old control and transgenic tobacco plants. The disks were floated in MS supplemented with NaCl (0 and 800 mM) for 3 days. Furthermore, total chlorophylls were isolated from leaf discs and determined as the quantity per gram of fresh tissue weight. The experiment was repeated with three biological replicates.

### Oxidative stress analyses

The 8-week-old plants of WT and OE lines were irrigated with 250 mM NaCl solution for one month, and other 8-week-old tobaccos were held without water for 15 days. Then, the leaves of WT and OE lines were detached for histochemical staining procedure. Staining was performed using DAB and NBT to detect the accumulation of H_2_O_2_ and O_2_^−^. A hydrogen peroxide test and a maleic dialdehyde assay kits (Nanjing Jiancheng Bioengineering Institute) were used to detect the H_2_O_2_ and O_2_^−^ concentrations and MDA contents. The enzymes were extracted in 5 mL of extraction buffer and the protein concentrations were quantified by BCA Protein Assay Kit (Nanjing Jiancheng Bioengineering Institute). The activities of the antioxidant enzymes SOD, POD and CAT were measured by the kits from Nanjing Jiancheng Institute. Each assay was repeated three times.

### Construction and generation of transgenic yeast

*NtLTP4* and *NtLTP4*^27–118,*aa*^ were cloned into pYES2 vector. Afterwards, transformation into Gold Yeast cells was performed according to the manufacturer’s instruction (Clontech). The yeast transformed with empty pYES2 vector was used as control. Transgenic yeast cells with a series of dilution gradient were planted on YPDA solid medium with various concentrations of NaCl and mannitol. Photographs were taken at 30 °C for 48 h. The transgenic strains were inoculated to the liquid medium containing different concentrations of NaCl and mannitol, and the absorbance values of the stock cultures were determined 48 h later. The absorbance values were obtained and reported as the value relative to YPDA corresponded to different stock cultures in YPDA liquid medium. Each assay was repeated three times.

### Yeast two-hybrid assay

Yeast transformation and screening were conducted following the manufacturer’s instructions (Clontech, USA). Yeast Gold cells were co-transformed with specific bait and prey constructs. All of the yeast transformants were grown on SD/-Leu/-Trp (DDO) and SD/-Leu/-Trp/-His/-Ade with X-a-gal (QDO) medium for selection or the interaction test.

### Co-IP

For Co-IP assays, *A*. *tumefaciens N*. *benthamiana* leaves were harvested to extract proteins with IP buffer (50 mM Tris-HCl pH 7.5, 150 mM NaCl, 0.1% NP-40, 5 mM DTT, protease inhibitor cocktail) as previously described^55^. The leaf extracts were centrifuged to obtain a supernatant and incubated with GFP and Protein A beads (Invitrogen) for 6–8 h at 4 °C. The beads were recovered from the mixture by centrifugation and washing three times with cold washing buffer. Total protein was separated in 10% SDS-PAGE gel for Western Blot analysis by using an HA antibody (Sigma, Shanghai, China) and Pierce ECL western blot substrate (Thermo Fisher Scientific, Rockford, IL, USA).

### LCI (firefly luciferase complementation imaging) assay

An LCI assay was performed following a previous study^56^. Different combinations of *A*. *tumefaciens* with related constructs were infiltrated into different positions in the same leaves of *N*. *benthamiana* for 60 h. Luciferin (Promega) was infiltrated into the leaves, and luciferase activity was measured with a low-light cooled CCD imaging apparatus (Lumina II, USA).

### Statistical analysis

All of the experiments in this study were conducted three times. Error bars in each graph indicate the mean values ± standard error (SE) of replicates. Statistically significant differences between measurements were determined using one-way ANOVA in IBM Software version 24 (IBM, USA).

## Electronic supplementary material

supplementary data
