# Supplementary material for: NtLTP4, a lipid transfer protein that enhances salt and drought stresses tolerance in Nicotiana tabacum
Source: Sci Rep. 2018 Jun 11;8:8873. doi: 10.1038/s41598-018-27274-8 (PMC5995848; doi:10.1038/s41598-018-27274-8)
Supplement: Supplementary file 1 — supplementary data [file 41598_2018_27274_MOESM1_ESM.pdf]

Published in *Scientific Reports*.

Title:

NtLTP4, a lipid transfer protein that enhances salt and drought stresses tolerance in *Nicotiana tabacum*

Yang Xu[1*], Xinxin Zheng[1*], Yunzhi Song[1], Lifei Zhu[1], Zipeng Yu[1], Liming Gan[1], Shumei Zhou[2], Hongmei Liu[1], Fujiang Wen[1], Changxiang Zhu[1#]

Affiliations:
[1]State Key Laboratory of Crop Biology, College of Life Sciences, Shandong Agricultural University, Tai′an, Shandong, P.R China
[2]State Key Laboratory of Crop Biology, College of Agronomy, Shandong Agricultural University, Tai′an, Shandong, P.R China
[*]Co-first Author: Yang Xu, Xinxin Zheng
[*]The authors contributed equally to this work.

[#]Corresponding author:
Changxiang Zhu
State Key Laboratory of Crop Biology, College of Life Sciences, Shandong Agricultural University, Tai′an, Shandong, P.R China
E-mail: zhchx@sdau.edu.cn

Fig. S1

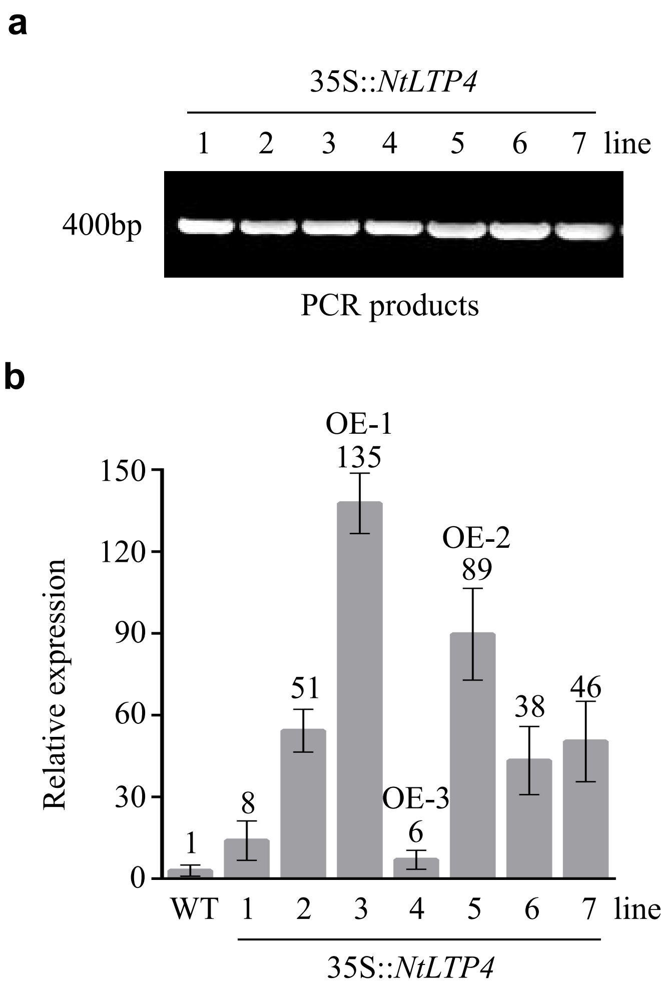

Supplementary Figure S1. The expression levels of NtLTP4 in overexpressing lines. **(a)** PCR identification of the transgenic tobacco plants. **(b)** RNA extracted from leaves of 14-day-old *N. tabacum* and qPCR analysis of *NtLTP4* expression levels in different overexpressing lines.

Fig. S2

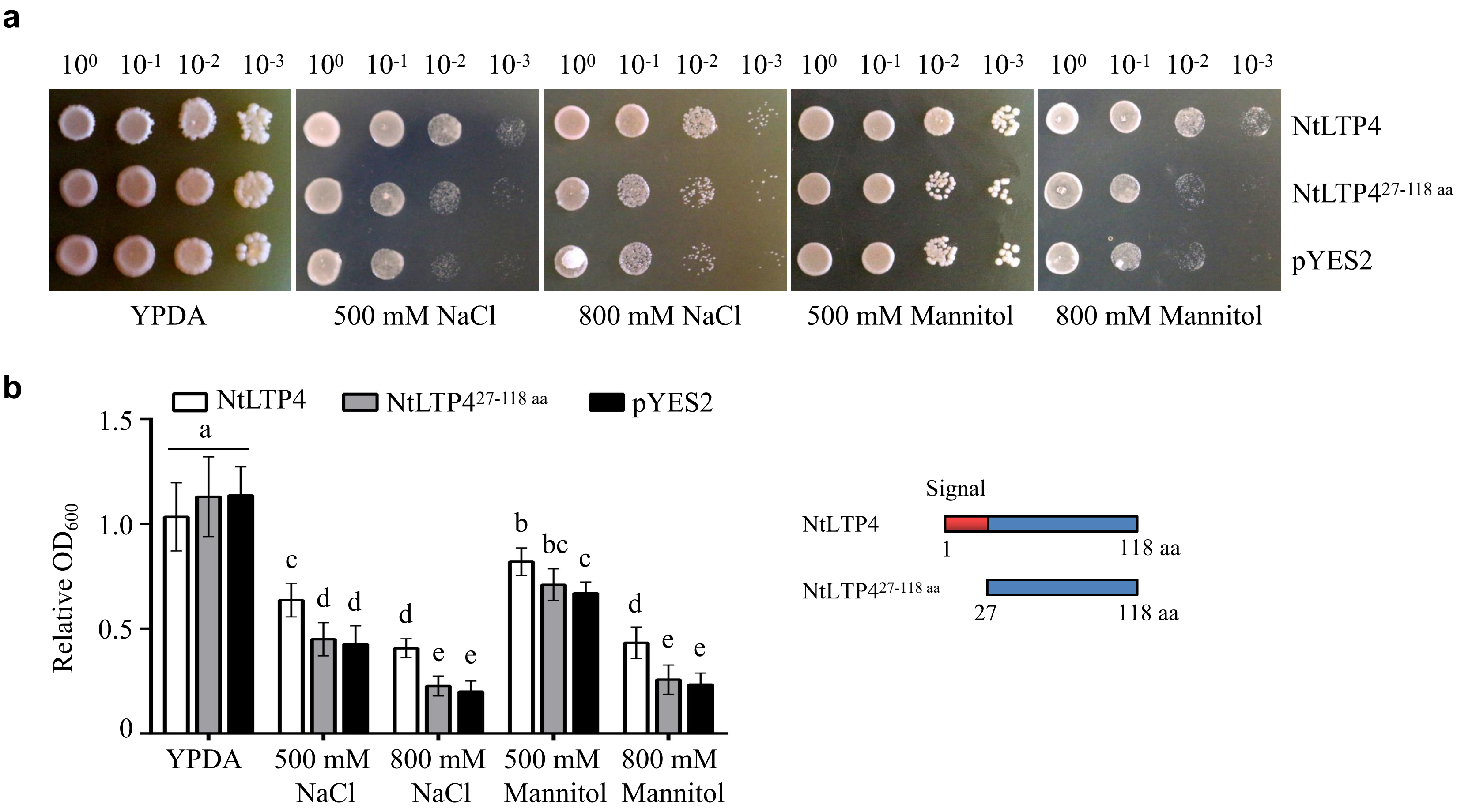

Supplementary Figure S2. Overexpression of *NtLTP4* in transgenic yeast enhanced salt and drought tolerance.
(a) Yeast transformants containing pYES2 empty vector, NtLTP4$^{27\text{-}118\ aa}$, or NtLTP4 cultured on YPDA solid medium with or without various concentrations of NaCl and Mannitol. **(b)** Absorbance of stock cultures at OD_{600} in YPDA liquid medium with or without various concentrations of NaCl and mannitol, which obtained and reported as value corresponding to different stock cultures in YPDA liquid medium. The structures of plasmids used for this experiment demonstrated in the right. The red cuboids indicate the signal peptide of NtLTP4; the blue cuboids indicate the likely functional domain. Experiment was repeated three times. Error bars indicate SEM. One-way ANOVA (Duncan′s multiple range test) was performed, and statistical significant differences are indicated by different lowercase letters ($P < 0.01$).

Fig. S3

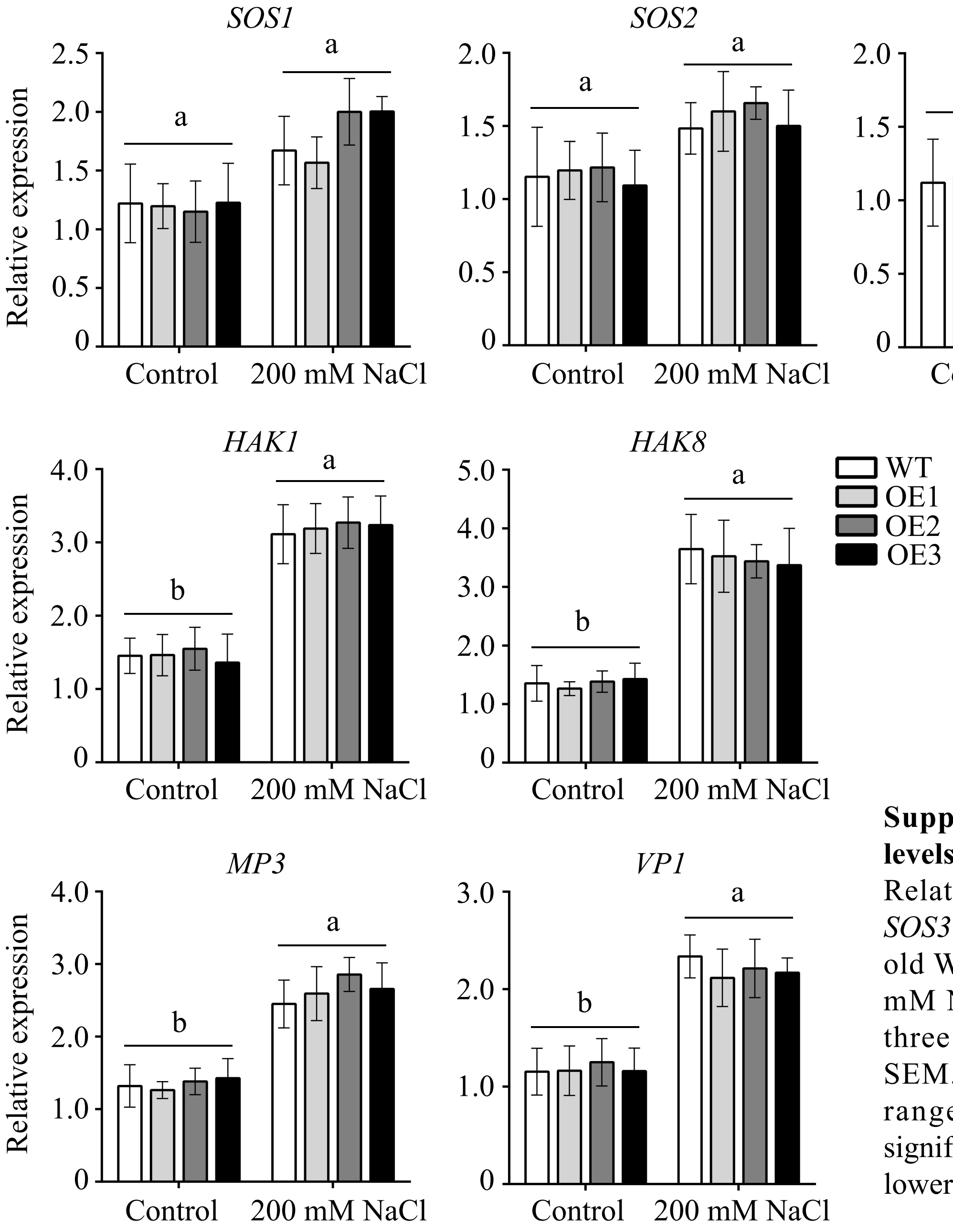

Supplementary Figure S3. The expression levels of Na^+ homeostasis associated genes. Relative expression analysis of *SOS1*, *SOS2*, *SOS3*, *HAK1*, *HAK8*, *VP1*, and *MP3* in 14-day-old WT and OE lines treated with MS or 200 mM NaCl for 48 h. Data as mean values of three biological repeats. Error bars indicate SEM. One-way ANOVA (Duncan′s multiple range test) was performed, and statistical significant differences are indicated by different lowercase letters (P< 0.01).

Fig. S4

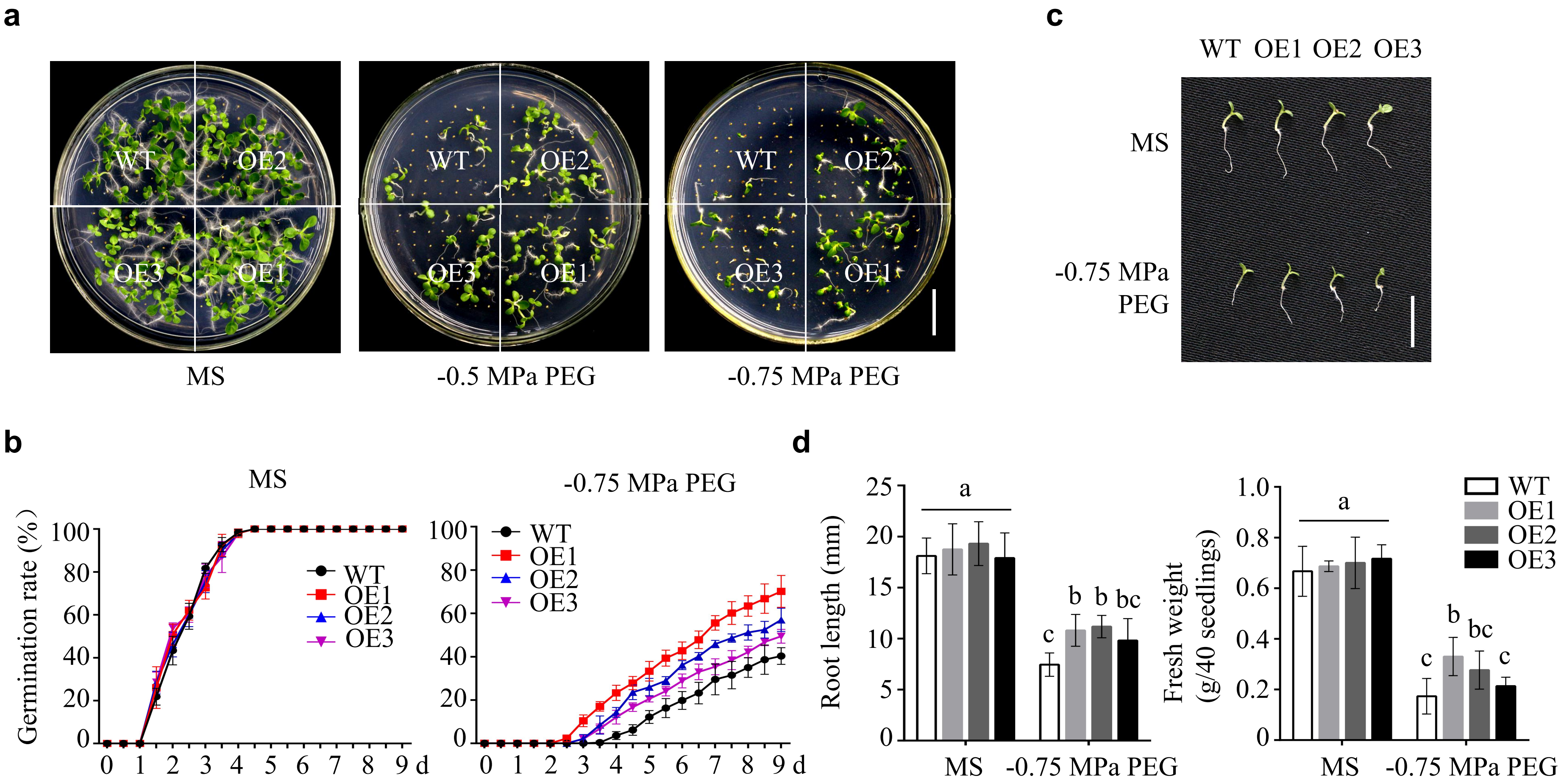

Supplementary Figure S4. Overexpression of *NtLTP4* in transgenic plants enhanced drought tolerance.
(a) Seed germination on MS medium containing various concentrations of PEG. Bar= 1 cm. **(b)** Germination rates of WT and OE lines in MS medium with or without PEG. **(c)** Seedling phenotype of 14-day-old WT and OE lines with or without PEG treatment. Bar= 1 cm. **(d)** Root length and fresh weight of seedlings shown in (c). Data as mean values of three biological repeats. Error bars indicate SEM. One-way ANOVA (Duncan′s multiple range test) was performed, and statistical significant differences are indicated by different lowercase letters (P< 0.01).

Fig. S5

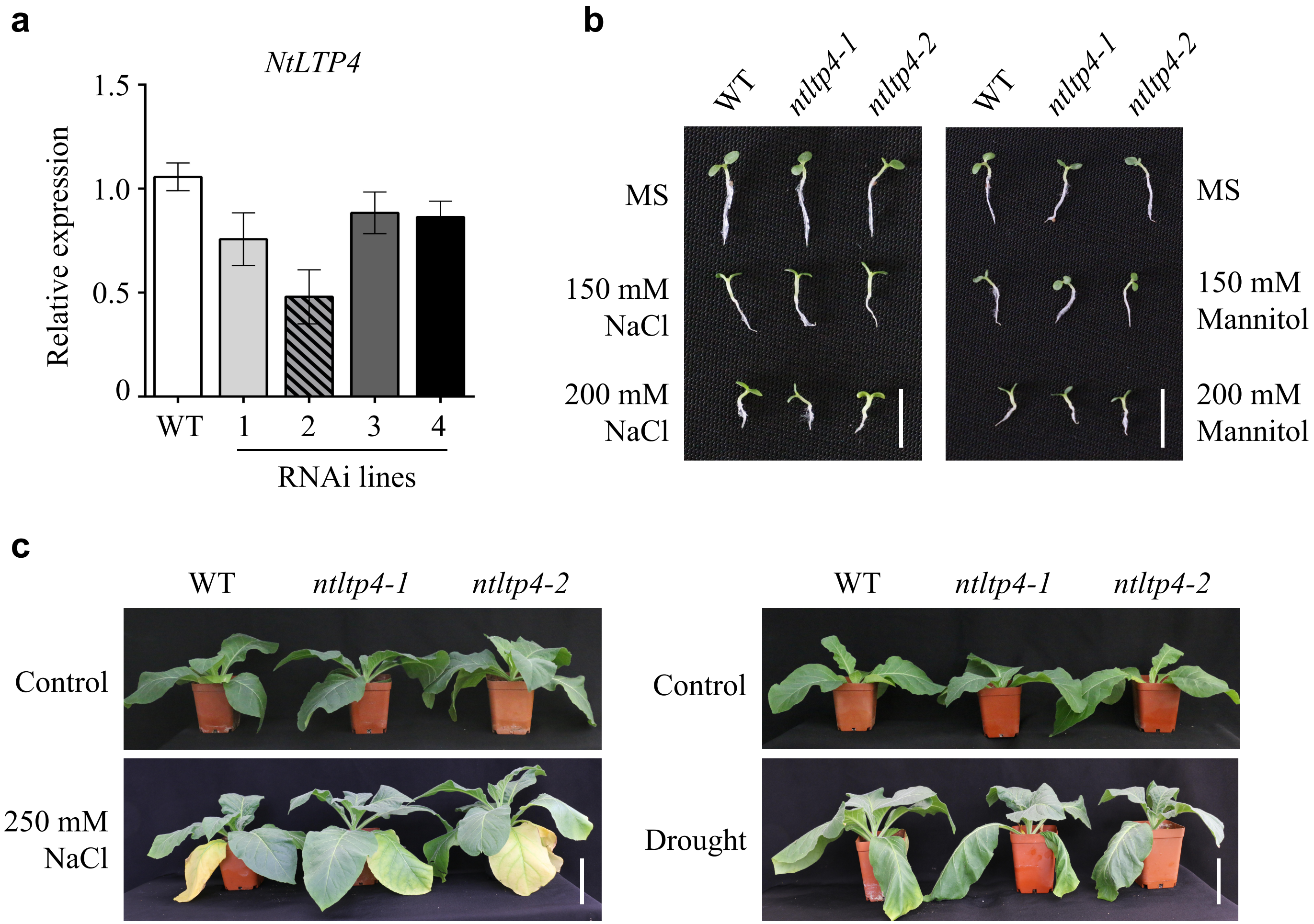

Supplementary Figure S5.The phenotype of RNAi lines under salt and drought stress.
(a) Relative expression analysis of *NtLTP4* in 14-day-old RNAi lines. **(b)** Seedling phenotype of 14-day-old WT and RNAi lines with or without salt and drought stress treatment. Bars= 1 cm. **(c)** Phenotype of 8-week-old WT and RNAi lines grown in soil irrigated with water and 250 mM NaCl solution for 1 month for salt stress treatment, and with or without dehydration treatment for 15 days for drought stress treatment. Bars= 2 cm.

Supplementary Table S1. Seven prey proteins screened by yeast-two-hybrid assay.

Annotation/locus ID	Description
LOC107791032	Nicotiana tabacum suppressor of mec-8 and unc-52 protein homolog 2-like
LOC107814725	Nicotiana tabacum large proline-rich protein BAG6
LOC107806366	Nicotiana tabacum plant intracellular Ras-group-related LRR protein 7-like
LOC107793888	Nicotiana tabacum ankyrin repeat domain-containing protein 2B-like
LOC107799413	Nicotiana tabacum uncharacterized LOC107799413
LOC107794718	Nicotiana tabacum uncharacterized LOC107794718
LOC107821857	WIPK, mitogen-activated protein kinase 3-like

Supplementary Table S2. Primers used in this study.

Purpose	Name	Sequence
qRT-PCR	β-Actin-F	TTGGCTTACATTGCTCTTG
	β-Actin-R	TCATTGATGGTTGGAACAG
	NtGST-F	GCTTGGGTTAAGGGATTG
	NtGST-R	AGACAACTACTTCAGACAGA
	NtCAT-F	TTCTCCTACTCTGATACC
	NtCAT-R	ATAGTGATTGTTGTGATGA
	NtAPX-F	ACTGCTAATCCACTTATCTT
	NtAPX-R	GTCTGATGGCAACTGTAA
	NtSOD-F	ACCAGAAGCATCATCAGA
	NtSOD-R	CTTTGGAAATGGCGTCAT
	NtRbohA-F	AAGGTGTTATGAACGAAGTG
	NtRbohA-R	CTGGTGCCTGATACGATA
	NtRbohB-F	CTATGCTTCAGTCTCTTCAC
	NtRbohB-R	GGCGTGTTGTCTTAGTTC
	NtSOS1-F	TGGAGGAAGCGACCGATTC
	NtSOS1-R	CGATAACGAGAAGAGCGACAG
	NtSOS2-F	CTGTTGCTCATTGTCACTG
	NtSOS2-R	CGTAACCCTGTCCACTAAG
	NtSOS3-F	GTCACGCCATTCACGGTAG
	NtSOS3-R	CACTCCATTTCGCTTCACATC
	NtNHX1-F	AAGAAGGTCATACTCAGTT
	NtNHX1-R	GGTAGCAATAGTCTAATCAAT
	NtHKT1-F	GTAATGATGTATCTTCCTCCAT
	NtHKT1-R	TTCTTCCACAGTCTCTTCT
	NtHAK1-F	AGATGTATAGCACGATATG
	NtHAK1-R	TTGTTCCAATAACTGTCA
	NtHAK8-F	AGATGTATAGCACGATATG
	NtHAK8-R	TTGTTCCAATAACTGTCA
	NtMP3-F	CTACTACACAGGACATCC
	NtMP3-R	AGCCATAAGAATACCAGAA
	NtVP1-F	ACTGGCACATATTCTTCTG
	NtVP1-R	CTGAACTGGACTGTAAGC

Purpose	Name	Sequence
Y2H assay	NtLTP4-AD-F	GAATTCATGGCTAAAGTAGCATTGTTGGTGG
Y2H assay	NtLTP4-AD-R	GGATCCTTACTGCACCGTGGAGCAGTC
Y2H assay	NtLTP4-BD-F	GAATTCATGGCTAAAGTAGCATTGTTGGTGG
Y2H assay	NtLTP4-BD-R	GGATCCTTACTGCACCGTGGAGCAGTC
Y2H assay	WIPK-AD-F	GAATTCATGGCTAATCAAATCGGCGGAGC
Y2H assay	WIPK-AD-R	GGATCCTCATATTTTTAGTTCAACAAAGTCTGGGTAGA
Y2H assay	WIPK-BD-F	GAATTCATGGCTAATCAAATCGGCGGAGC
Y2H assay	WIPK-BD-R	GGATCCTCATATTTTTAGTTCAACAAAGTCTGGGTAG
Transgenic yeast	NtLTP4-pYES2-F	GGATCCATGGCTAAAGTAGCATTGTTGGTGGTGTG
Transgenic yeast	NtLTP4-pYES2-R	GAATTCTTACTGCACCGTGGAGCAGTC
Transgenic yeast	NtLTP4-pYES2$^{27\text{-}118aa}$ -F	GGATCCATCTCTTGTGGGCAGGTTGTTGC
Transgenic yeast	NtLTP4-pYES2$^{27\text{-}118aa}$ -R	GAATTCTTACTGCACCGTGGAGCAGTC
Co-IP	WIPK-121-HA-F	GGATCCATGTACCCATACGACGTCCCAGACTACGCTGCTAATCAAATCGGCGGAGC
Co-IP	WIPK-121-HA-R	GAGCTCTCATATTTTTAGTTCAACAAAGTCTGGG
Co-IP	NtLTP4-121-GFP-F	GGATCCATGGCTAAAGTAGCATTGTTGGTGG
Co-IP	NtLTP4-121-GFP-R	GTCGACCTGCACCGTGGAGCAGTC
Transgenic plants	LTP OE F	TCTAGAATGGCTAAAGTAGCATTGTTGG
Transgenic plants	LTP OE R	GAGCTCTTACTGCACCGTGGAGCAGTC
Transgenic plants	LTP-GFP-F	GGATCCATGGCTAAAGTAGCATTGTTGGTGGTG
Transgenic plants	LTP-GFP-R	GGTACCCTGCACCGTGGAGCAGTC
Transgenic plants	LTP-RNAi-1-F	TCTAGATGGCTAAAGTAGCATTGTTGG
Transgenic plants	LTP-RNAi-1-R	GGATCCTTACTGCACCGTGGAGCAGTC
Transgenic plants	LTP-RNAi-2-F	GGTACCTGGCTAAAGTAGCATTGTTGG
Transgenic plants	LTP-RNAi-2-R	GGATCCGCAGATTTAATACAGTTACAAGCCGTCTG
LCI assay	LTP-cLUC-F	GGTACCATGGCTAAAGTAGCATTGTTGGTGG
LCI assay	LTP-cLUC-R	GTCGACTTACTGCACCGTGGAGCAGTC
LCI assay	LTP-nLUC-F	GGTACCATGGCTAAAGTAGCATTGTTGGTGG
LCI assay	LTP-nLUC-R	GTCGACTTACTGCACCGTGGAGCAGTC
LCI assay	WIPK-cLUC-F	GGTACCATGGCTAATCAAATCGGCGGAGC
LCI assay	WIPK-cLUC-R	GTCGACTCATATTTTTAGTTCAACAAAGTCTGGGTAGA
LCI assay	WIPK-nLUC-F	GGTACCATGGCTAATCAAATCGGCGGAG
LCI assay	WIPK-nLUC-R	GTCGACTATTTTTAGTTCAACAAAGTCTGGGTAGA

The original figures

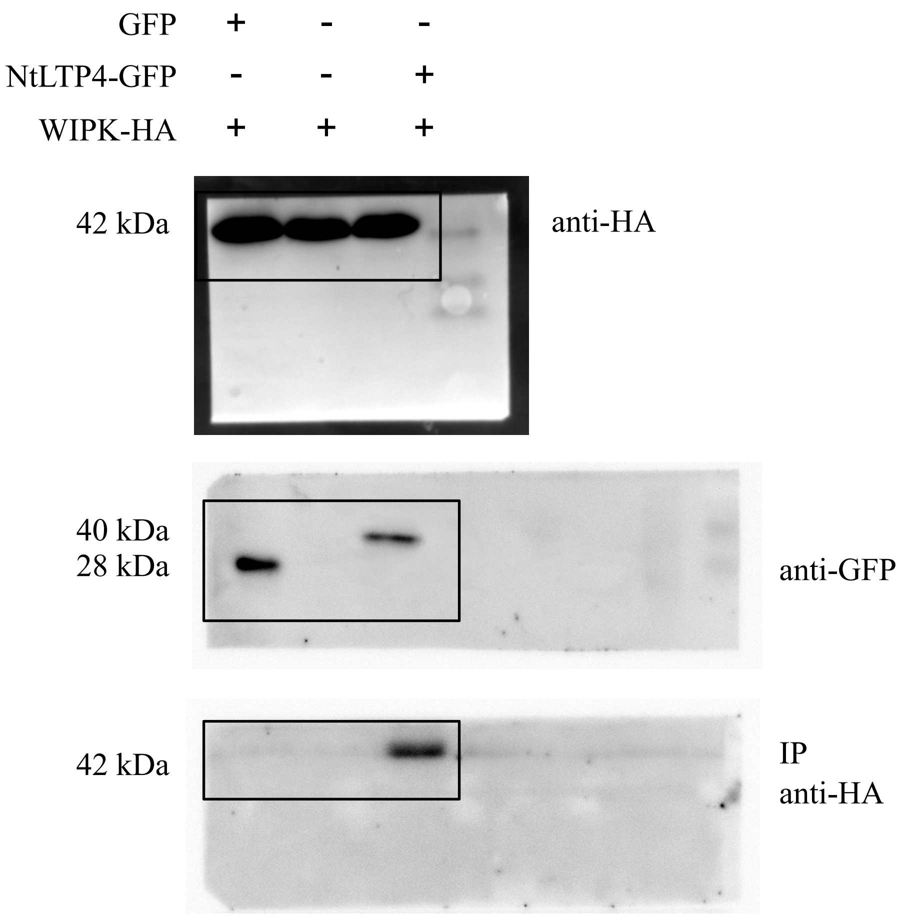

Images showed in Figure 8e are indicated by black boxes.

The original figure

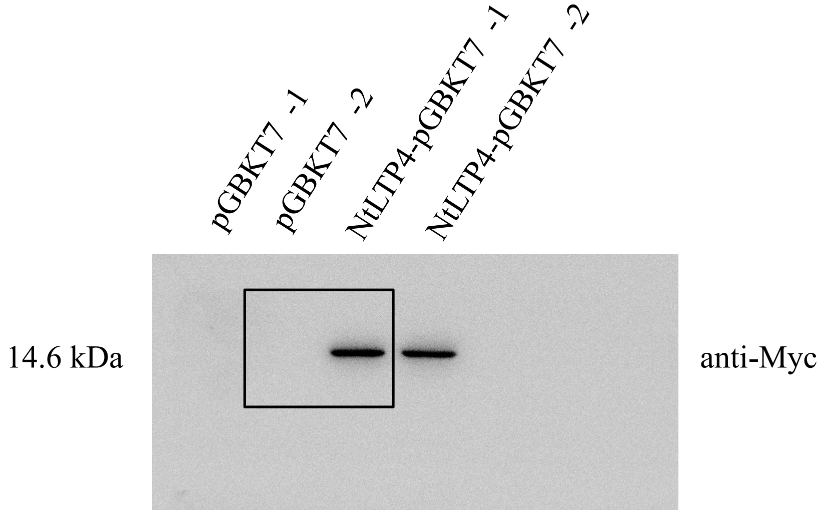

Image showed in Figure 8a is indicated by black box.
